# The Association of Autonomic Nervous System Function With Ischemic Stroke, and Treatment Strategies

**DOI:** 10.3389/fneur.2019.01411

**Published:** 2020-01-22

**Authors:** Mengxi Zhao, Ling Guan, Yilong Wang

**Affiliations:** ^1^Department of Neurology, Beijing Tiantan Hospital, Capital Medical University, Beijing, China; ^2^National Clinical Research Center for Neurological Diseases, Beijing, China; ^3^Department of Neurology, China National Clinical Research Center for Neurological Diseases, Beijing Tiantan Hospital, Capital Medical University, Beijing, China; ^4^Advanced Innovation Center for Human Brain Protection, Capital Medical University, Beijing, China

**Keywords:** autonomic nervous system function, heart rate variability, stroke, risk stratification, outcome, intervention

## Abstract

Acute ischemic stroke, especially minor stroke, and transient ischemic attack have high risks of recurrence and exacerbation into severe ischemic strokes. It remains challenging to perform risk stratification and screen high-risk groups for initiation of early treatment in these patients. Moreover, with the growing population of patients with chronic small vessel disease, the mechanisms and clinical implications require further investigation. Traditional tools such as the ABCD2 score (age, blood pressure, clinical features, duration of symptoms, diabetes) have only moderate predictive value in patients with transient ischemic attack or minor stroke. By contrast, measurement of changes in heart rate variability (HRV) is an important and novel tool for risk stratification and outcome prediction in patients with cardiovascular diseases, as it reflects the overall level of autonomic nervous system dysfunction. Thus, abnormal HRV may be useful for prognosis and improve stratification of stroke patients with diverse risks. HRV may also partially explain autonomic nervous dysfunction and other manifestations during the process of chronic cerebral small vessel disease. In summary, measurement of HRV may contribute to early initiation of interventions in acute or chronic stroke patients using novel treatments involving rebalancing of autonomic nervous system function.

## Introduction

Neurological disorders are the main contributor to disability-adjusted life-years, for which stroke is largest component (42.2% [38.6–46.1%]) (1). The risk of recurrent stroke is ~10–20% in patients with minor stroke or transient ischemic attack (TIA) (2–4). A recent analysis showed that stroke patients had ~14.5% risk of developing secondary ischemic events within 5 years in low-and middle-income countries (5). Risk stratification and identification of high-risk individuals for initiation of early treatment in these patients remains challenging. Traditional tools such as the Age, Blood pressure, Clinical features, Duration of symptoms, and Diabetes (ABCD2) risk score have only moderate predictive value of recurrence at 7 days after minor stroke [area under the curve (AUC) 0.64; 0.53–0.74; *P* = 0.03] covering limited risk factors or body stressors.

Organisms respond to various stressors and conditions, where an unpredictable and uncontrollable environmental demand exceeds the natural regulatory capacity to rebuild homeostasis, by regulation of the autonomic nervous system (ANS) (6). Heart rate variability (HRV) is thought to reflect the overall stresses acting on the body, as it is a precise and valid measurement of ANS. Importantly, impaired ANS with decreased HRV is associated with known risk factors for stroke, which likely results in development of severe ischemic events (7, 8) and a worse prognosis (9, 10).

Apart from acute cerebrovascular diseases, chronic small vessel disease presenting as white matter lesions and other abnormalities on brain imaging is also prevalent, particularly in older adults (11). The clinical manifestation includes acute focal neuro-dysfunction, as well as cognitive dysfunction, gait and balance problems, mood disorders, and autonomic dysfunction (12). The mechanism is complex, and the relationship between chronic cerebrovascular disease and ANS remains unclear.

This review article summarizes the relationship between ANS function and acute or chronic cerebrovascular disease, the utility of HRV for risk stratification and prognosis of acute ischemic stroke (AIS), and possible therapeutic interventions for stroke based on restoration of sympathetic-vagal balance.

## Acute Ischemic Stroke and TIA

According to the ranking of nationwide cause of death published by the Chinese Ministry of Health in 2004, cerebrovascular disease ranks only second to cancer, and is the main cause of disability and death in the urban and rural population (13). AIS, caused by abnormal cerebral perfusion because of sudden rupture or occlusion of the cerebral vasculature, is the most common type of stroke, accounting for 60–80% of all stroke patients (14, 15). In addition, the China National Stroke Registry II reported a high mortality in AIS patients, with an in-hospital and 3-month mortality of 1.1 and 4.8%, respectively (16). Despite non-disabling or temporary symptoms (2, 3), ~10–20% of patients have another stroke secondary to a TIA or minor stroke within 3 months (4). The main cause of the high incidence and mortality of cerebrovascular disease in China may relate to the increasing number of people with stroke risk factors (13, 17). Thus, risk stratification and identification of patients at high risk of imminent stroke is important.

Traditional prediction tools such as the ABCD2 score are widely implemented to evaluate the risk of recurrent ischemic events in patients with TIA or minor stroke (18). However, they have only moderate predictive value of recurrence at 7 and 90 days after TIA or minor stroke (AUC 0.55–0.7) (8, 18). Other validated clinical risk prediction tools, including the Essen Stroke Risk Score and the Stroke Prognosis Instrument II, are only used to predict the long-term risk of recurrence after TIA or minor stroke, and neither of these tools are predictive of the early risk of stroke recurrence (19). Therefore, a new, reliable, and convenient early risk prediction tool is required.

## ANS, Stress, and HRV

### ANS, Stress, and Health

The ANS, composed of the sympathetic nervous system (SNS) and the parasympathetic nervous system (PNS), is usually controlled by the sympathetic-vagal balance (8, 20, 21). The ANS also regulates initiation of the body's stress response to various stressors perceived by the brain, including acute and chronic risk factors for stroke, to neutralize the effects of the stressors and restore homeostasis (6, 22). However, when the demands for restoration of homeostasis exceed the adaptive capacity, impaired ANS function caused by sympathetic-vagal dysregulation can result in inaccurate responses or progressive delayed reactivity, ultimately causing stress-related disorders such as cerebrovascular disease (6).

### HRV for Assessing ANS Function

Measurement of HRV is an established and widely-implemented tool for assessing ANS function and risk stratification in patients with cardiovascular disease (8, 20, 23). HRV parameters obtained by either short-term 5-min recordings or nominal 24-h long-term recordings of a continuous electrocardiographic record (24). include the standard deviation of the NN intervals (SDNN), and the root mean square successive difference of intervals (RMSSD). Frequency domain parameters include high-frequency (HF; range 0.15–0.4 Hz), low-frequency (LF; range 0.04–0.15 Hz), very low-frequency (VLF; <0.04 Hz), and the ratio of LF to HF power (LF/HF) (20, 23). SDNN reflects comprehensive ANS function. RMSSD and HF represent the level of PNS activity. LF is modulated by both the SNS and PNS. Moreover, LF/HF reflects sympathetic-vagal balance (8, 20). Another novel complexity measurement of HRV called multiscale entropy can analyze the complexity of non-linear and non-stationary signals (25). Thus, ANS dysfunction and unstable sympathetic-vagal balance secondary to various stressors can cause changes in the normal HRV parameters (8, 20).

## ANS and HRV in AIS and Chronic Cerebrovascular Disease

### Relationship Between HRV and Stroke Risk Factors

**Figure 1:**
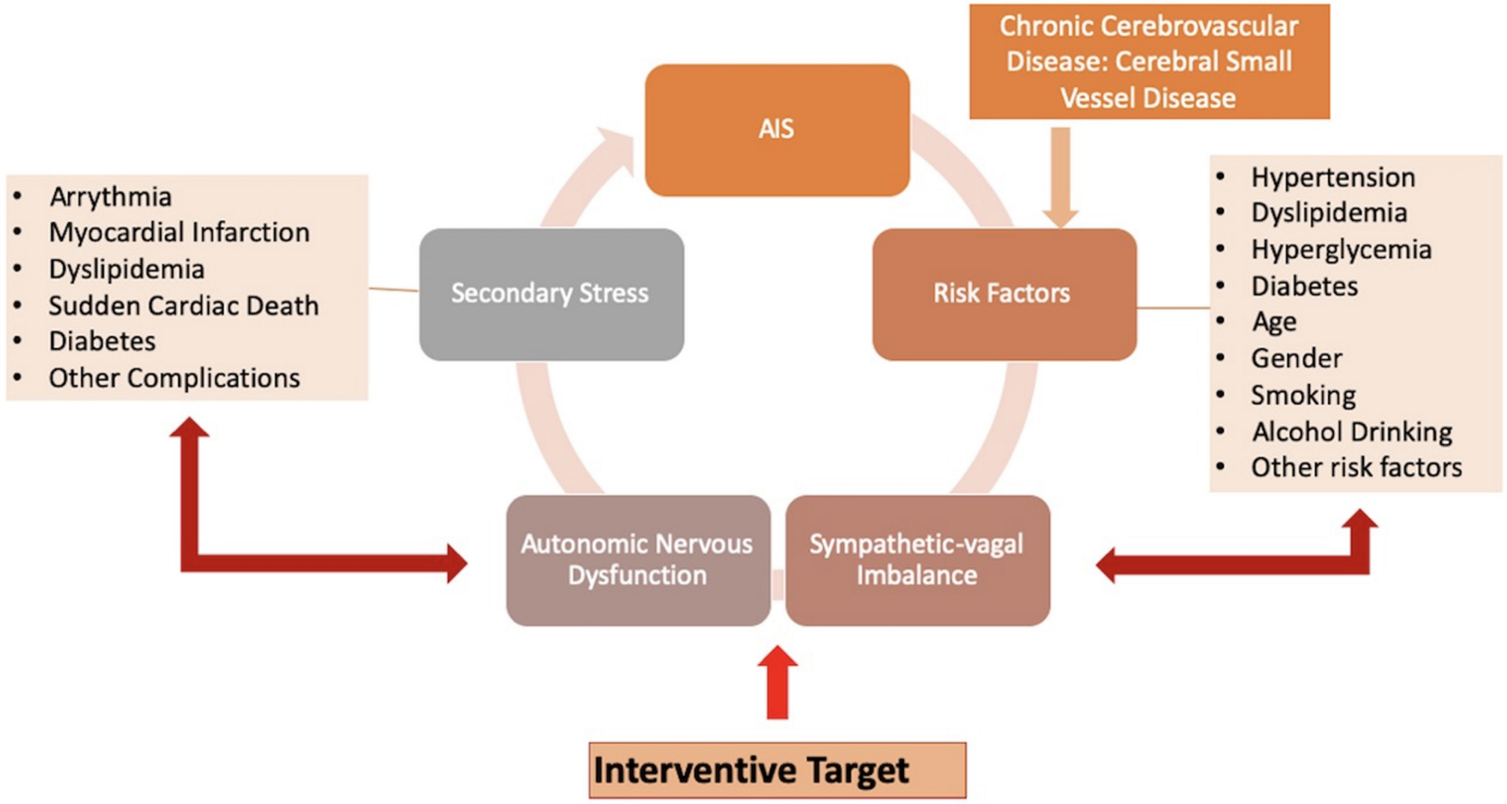
Hypertension, diabetes, smoking, and other risk factors of stroke, as well as acute ischemic stroke (AIS) and cerebral small vessel disease (CSVD), may have a negative influence on the body. Regulated by the autonomic nervous system (ANS), the body initiates the stress response to these various stressors perceived by the brain, in an attempt to neutralize the effects of the stressors and regain homeostasis. When demands for re-establishment exceed the adaptive capacity, impaired ANS attributed to altered sympathetic-vagal balance may provide inaccurate responses or progressively lead to a delayed reactivity. This eventually evolves into stress-related disorders as secondary stresses, such as arrhythmia, myocardial infarction, dyslipidemia, and other complications. These secondary stressors increase the incidence of recurrent ischemic stroke. These pathways can form a vicious cycle, while targeting sympathetic-vagal imbalance maybe an efficient interventional strategy.

Metabolic disorders such as hypertension, hyperlipidemia, and hyperglycemia are major risk factors for stroke, while ANS dysfunction is associated with these risk factors. SNS hyperactivity was reported to play a crucial role in patients with hypertension (26). An abnormal HRV is strongly related to individual blood pressure (BP) regulation and occurrence and development of hypertension (27). Diabetes is also associated with ANS dysfunction, which is mainly explained by a relative increase in SNS activity. Inflammatory or stress responses caused by impaired ANS function play a critical role in the development and progression of diabetes (28). A negative effect of elevated blood glucose levels on cardiac autonomic function was also reported, with an association of the duration of hyperglycemia or diabetes with the degree of decrease in HRV (29). Researchers also found that patients with both diabetes and hypertension showed lower HRV values, suggesting that hypertension may have a negative effect on cardiac autonomic function in patients who are already prone to developing autonomic dysfunction (30, 31). An updated review delineated a reciprocal role of autonomic dysfunction and glucose metabolism abnormalities in a very early stage of dysglycemia, which need to be further elucidated (32). Dyslipidemia is another metabolic disorder. Elevated serum low density lipoprotein levels and hypercholesterolemia were reported to be associated with decreased HRV in patients with and without myocardial infarction or left ventricular dysfunction, suggesting that all dyslipidemia subjects had ANS dysfunction and an increased risk of sudden cardiac death (33). HRV is also associated with age, gender, smoking, lifestyle, alcohol drinking, and other stroke risk factors [Figure 1; (34, 35)].

### HRV and Infarction Characteristics

Since some brain structures (36) including anterior cingulate cortex, insular lobe, are reported significantly associated with cardiovascular function, cortical modulation of autonomic nervous function presents hemispheric lateralization in stroke patients (37). A prospective study enrolled 103 patients with right-sided infarction and left-sided infarction (38). Although all subjects had lower HRV parameters, the right-sided insular damage was significantly related to lower frequency domain parameters values and a higher LF/HF ratio. Chen et al. also measured HRV of 75 AIS patients divided into right hemispheric infarction, left hemispheric infarction, brainstem infarction, and control groups (39). Compared with controls, the AIS subgroups showed lower HF, LF, and HF% (HF power in normalized units), and elevated LF% (LF power in normalized units) and LF/HF, while the brainstem infarction group showed a distinct increase in sympathetic activity and a decrease in parasympathetic activity. These data provide evidence of a marked impairment of the cardiovascular ANS after AIS onset, and a correlation of HRV damage with infarct sites.

Another study evaluated the correlations of ANS function with early stroke outcome by measuring the HRV and the National Institutes of Health Stroke Scale (NIHSS scores) at the seventh day after admission in patients with different subtypes of acute stroke, including large artery atherosclerotic infarction (LAA) and lacunar infarction (LAC). Compared with the LAC and control groups, the LAA group had worse ANS dysfunction presenting as lower HF, lower normalized HF, higher normalized LF, and a higher LF/HF ratio, suggesting an association of reduced parasympathetic modulation with poorer early outcome in LAA patients (40). Patients with LAC were also reported to show a lower natural logarithm of HF and a higher LF/HF in the acute phase compared with those with LAA, indicating a higher risk of autonomic dysfunction in LAC patients in the acute phase of non-cardioembolic ischemic stroke (41). Furthermore, a recent review provided evidence suggesting a role for ANS dysfunction in the pathogenesis of cryptogenic stroke, with ANS-related alterations presenting as a sympathovagal imbalance leading to structural atrial changes and atrial cardiopathy (42).

### Relationship Between ANS Dysfunction and Outcomes of Stroke Patients

Dysfunctional autonomic responses after exposure to various stressors can impact on the corresponding target issues to generate multiple stress-related disorders, including changes in BP, pathoglycemia, and dyslipidemia. These stress-related disorders act as secondary stressors to further impair the sympathetic-vagal balance, which progressively increases the risk of cardiovascular and cerebrovascular diseases. The accumulation of stresses and potentiation of the pathogenic actions of the initial and secondary stressors forms a vicious cycle that can worsen outcomes [Figure 1; (8)]. As an indicator of overall stress levels in the entire body, HRV may be particularly useful for risk stratification and prediction of outcome in patients with cardiovascular and cerebrovascular diseases.

#### ANS Dysfunction, Post-stroke Disorders, and Outcomes

**Figure 2:**
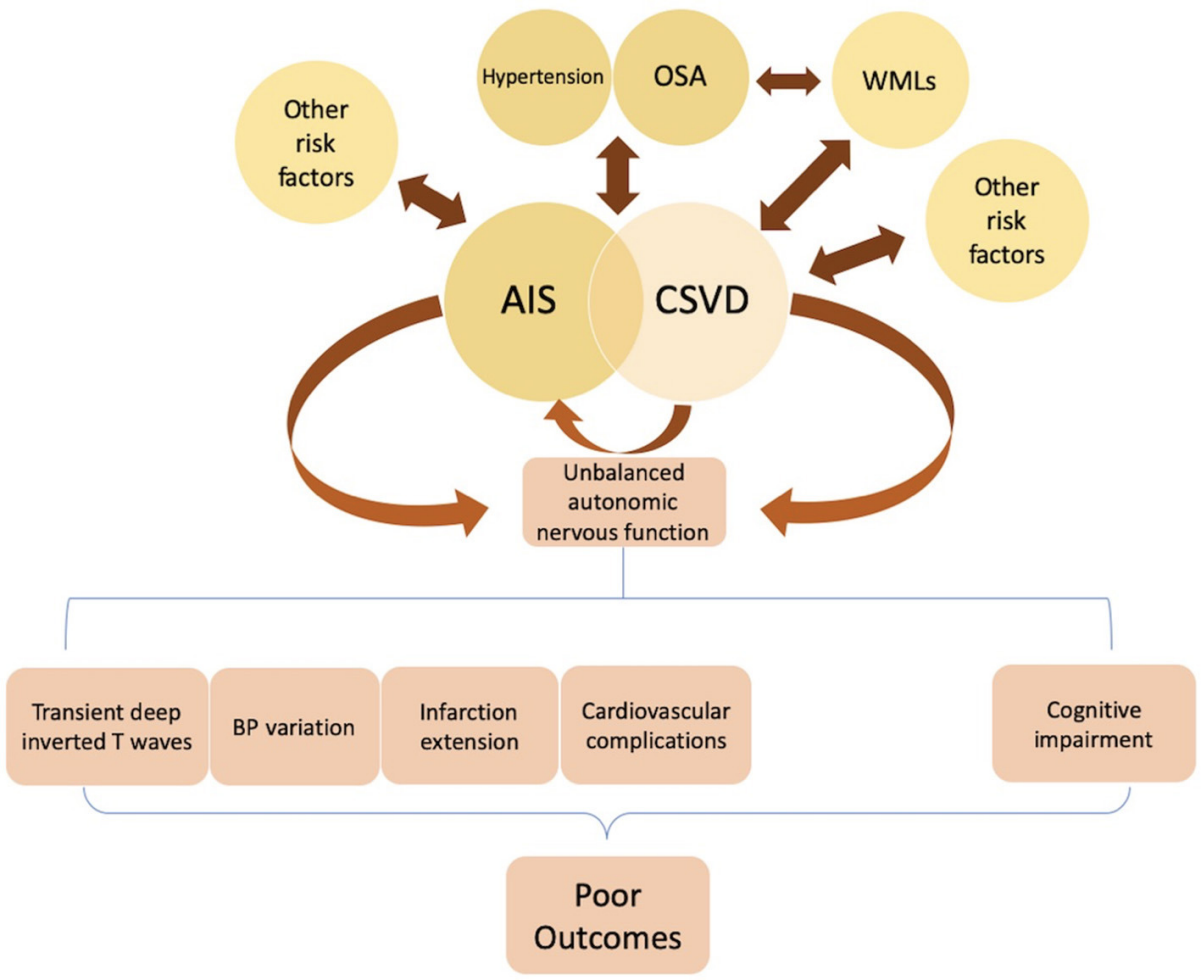
Hypertension, obstructive sleep apnea (OSA), smoking, and other risk factors are significantly associated with AIS. Studies found several pathologic changes after AIS onset, including transient changes in cardiac electrophysiology, blood pressure (BP) variation, and a higher probability of extensive infarction and in-hospital cardiovascular complications. These post-stroke deteriorations attributed to ANS dysfunction (i.e., sympathetic over-activity) may lead to poor clinical outcomes. Hypertension, OSA, and white matter lesions (WMLs) are considered independent risk factors of cerebral small vessel disease (CSVD). Some CSVD lesions present with acute stroke symptoms, while others are asymptomatic. A possible mechanism for these silent lesions may relate to locations in the motor or sensory tracts such as the internal capsule. Some studies suggest that dysregulation of heart rate variability (HRV), especially over-activation of sympathetic tone, may be a pathophysiologic mechanism underlying the development of WMLs in OSA patients. Failed autonomic control of the cerebral circulation can predispose patients to CSVD, leading to cognitive impairment, in which sympathetic activation may have a major role. Further studies examining the underlying pathogenesis of CSVD are required.

ANS dysfunction is strongly linked to poor prognosis in stroke patients (43). Rare, transient, symmetric, and deep inverted T waves were also reported in the electrocardiogram of AIS patients, which was considered to reflect a transient cardiac dysfunction after AIS (44). ANS dysfunction at the acute stage of cerebrovascular events may influence BP adjustment (Figure 2). For example, a study examining BP variation at the acute phase of first-ever stroke found a significant difference between stroke subtypes, and the higher the 24-h rate of systolic BP (SBP) variation, the worse the 1-year outcome. Furthermore, with each 0.1 mmHg/min increase in the 24-h rate of SBP variation, the odds of a negative outcome increased 1.96-fold (95% confidence interval: 1.16–3.32) (45). ANS dysfunction following acute cerebrovascular events may primarily relate to central ANS network damage. A recent study of sympathetic-vagal balance in patients with AIS showed significant differences in sympathetic activity measured by HRV parameters during early mobilization between patients with and without neurological deterioration, while there was no variation in BP, HR, or parasympathetic activity. This phenomenon may be explained by an increase in SNS activity during mobilization, leading to neurological deterioration (46). The lack of reliable tools for early stroke detection is a key reason for the low proportion of AIS patients who receive reperfusion therapy. In a rat model of middle cerebral artery occlusion (MCAO), Kodama et al. reported that assessment of HRV parameters immediately after ischemic stroke onset showed a high sensitivity (82%) and specificity (75%) for ischemic stroke detection, suggesting an potential ischemic stroke detection algorithm for human as a clinical tool (47).

Stroke-induced immunodepression is one of the characterized complications of AIS (48). Strokes involving the ANS-related brain structures increased the risk for pneumonia (OR 4.55, 2.41–8.56, *p* < 0.001) and sepsis (OR 4.13, 1.81–9.43, *p* = 0.001) (49). VLF was found able to predict infection from days 3 to 5 after AIS onset with AUC = 0.80 (cross-validation AUC = 0.74) after adjustment for NIHSS at admission and diabetes (50). Additionally, these studies suggested that HRV analysis starting immediately after admission may assist identifying patients at risk of post-stroke infection.

With respect to the prognostic evaluation of HRV, Xiong et al. stratified AIS patients on the basis of the magnitude of autonomic dysfunction measured within 7 days from symptom onset using the Ewing's battery test to estimate the 3-month modified Rankin Scale score. In that study, patients with significant impairment of autonomic function had worse functional outcomes compared with those with minor autonomic impairment, suggesting that post-stroke autonomic dysfunction can predict poor clinical outcome (10). Nayani et al. also examined autonomic function at 2–4 weeks recovery in first ever ischemic stroke patients using the Ewing's battery test and 24-h Holter analysis for HRV, while neurological and cardiovascular outcomes were evaluated at discharge, 3 months, and 1 year after stroke onset (9). Patients with significant autonomic dysfunction had more severe stroke at discharge, a higher probability of extensive infarction and in-hospital cardiovascular complications, and a poorer outcome at 1 year, which were independent of onset severity, age, hemispheric laterality, or presence of comorbidities. Overall, these studies suggest that autonomic function measured by HRV, representing the overall adjustment to a stress response, may be useful for predicting post-stroke clinical outcomes.

#### Use of HRV for Stratifying the Risk of Stroke

HRV has been applied in multiple risk stratification models according to a few studies and has showed its advantage. The HRV-based classifier could identify hypertensive patients at high risk of developing vascular events with high sensitivity and specificity (71.4 and 87.8%, respectively) (51). A combination model incorporating HRV and other disease severity score variables showed optimal predictive ability of 30-day in-hospital mortality for septic patients at the emergency department against conventional risk stratification tools (AUC = 0.91, 95% confidence interval: 0.88–0.95) (52, 53). The exploitation of HRV in risk stratification tools of stroke becomes one of the vital and prophylactic measures for high-risk individuals to ring alarm bells.

Arteriosclerosis is a major mechanism involved in the pathogenesis of ischemic stroke. Carotid arteriosclerotic disease is the main manifestation of generalized arteriosclerotic disease (54). A higher carotid intima–media thickness indicates a higher risk of incident stroke, as values >1.15 mm were reported to significantly increase the risk of stroke 19-fold (55). A recent study also demonstrated that carotid stenosis severity was inversely associated with vagal activity measured as reduced HRV (56). These data suggest that HRV may be useful for detecting high risk subjects who may benefit most from surgical or revascularization procedures.

In the Atherosclerosis Risk in Communities study, HRV was measured in 12,550 middle-aged adults, and Cox regression analysis showed a significant and strong association of reduced HRV parameters with a modest risk of incident stroke in adults with diabetes mellitus, irrespective of traditional cardiovascular risk factors (57). These previous studies also demonstrated that decreased HRV precedes poor outcome, suggesting that low HRV may be an early indicator of declining health in populations already at high risk.

In a large observational cohort study of 5,000 community-resident adults followed up for average 4.6 years, researchers found elevated RMSSD strongly associated with a higher risk of cerebrovascular events (HRs = 1.91–2.28). While the reclassification for 5-year cerebrovascular risk among those without prior AF diagnosis improved 14% (95% confidence interval: 7–21%) (58).

In another study examining risk stratification evaluation for HRV in 884 stroke-free participants, 68 stroke patients were observed over a median 7.3 years follow-up (7). Although high-risk identification using the Cardiovascular Health Study clinical stroke risk score was confirmed (the c-statistic for the new model was 0.61), their new model using a combination of HRV parameters had a significantly higher screening value for high-risk patients (the c-statistic for the new model was 0.68, *P* = 0.02), suggesting that HRV was important for stratifying stroke patients with high risks.

Although multiple studies have demonstrated HRV being associated with a range of risk factors for stroke and being predictive of cardiac and all-cause mortality, clinicians still have inadequate guidance in finding diagnostic reference levels in HRV that could indicate further elevated risk. Jarczok et al. provided the first evidence that daytime RMSSD below 25 ± 4 ms may be associated with elevated risk across a range of established cardiovascular risk factors [odds ratios (OR) 1.5–3.5]. The study suggested that HRV was robustly associated with a measure of self-rated health than a range of other biomarkers (59). Additional research is needed to validate clinical cut-off values for HRV. Thus, the extent of HRV disruption may be valuable for stroke risk stratification and targeting high-risk patients.

The relationship between HRV and stroke was described from three aspects in sections Relationship Between HRV and Stroke Risk Factors, HRV and Infarction Characteristics, and Relationship Between ANS Dysfunction and Outcomes of Stroke Patients: stroke risk factors, stroke characteristics (including infarct locations and pathogenesis), and stroke outcomes (including functional prognosis, complications, and future events). The involvement of ANS dysregulation is revealed in both the occurrence and development of stroke, which was implicated as both the cause and the consequence (60). ANS dysfunction is closely related to stroke.

### HRV and Chronic Cerebrovascular Disease

In addition to ischemic stroke caused by acute arterial occlusion, cerebral small vessel disease (CSVD) is a unique disorder of the cerebral microvessels, and has a chronic course and characteristic white matter lesions (WMLs) on brain imaging (61). Many CSVD patients have an asymptomatic manifestation, although progressive brain damage can lead to stroke, cognitive dysfunction, gait and balance problems, mood disorders, and urination and defecation disorders in older patients (12). An association of ambulatory 24-h HR with advanced WMLs was reported in subacute ischemic stroke patients, with a high 24-h HR (odds ratio = 1.041; 95% confidence interval = 1.006–1.078; *P* = 0.023) independently related to advanced WMLs, in addition to old age and high 24-h SBP levels (62).

Obstructive sleep apnea (OSA) is also a risk factor for stroke (63) and is associated with cerebral WMLs, while moderate-to-severe OSA (apnea-hypopnea index ≥ 15) was positively associated with CSVD (64). Using non-linear HRV indices, over-activation of sympathetic tone was suggested to represent a pathophysiologic mechanism for development of WML in OSA patients (65). Furthermore, failed autonomic control of cerebral circulation because of hypertension was reported to predispose individuals to CSVD and cognitive impairment, in which sympathetic activation may play a major role [Figure 2; (66)]. In a cohort study of 190 community-dwelling older adults in Japan, the nighttime RMSSD was independently correlated with the progression of CSVD (1 beat per min increase: odds ratio = 1.13; 95% confidence interval = 1.04–1.24; *P* < 0.01). Moreover, that study showed an independent association of HRV in the daytime and 24-h HR with cognitive decline (*P* < 0.05). Finally, increased nighttime HRV was reported to be a predictor of CSVD progression (67). Although further studies are required to fully understand the underlying pathogenesis of CSVD, it is clear that ANS function plays an important role.

Nearly 25% of patients presenting with ischemic stroke have lacunar or small vessel stroke (11). However, it remains unclear why some CSVD lesions present with acute stroke symptoms, while others are asymptomatic. A potential mechanism relates to the presence of lesions affecting the motor or sensory tracts such as the internal capsule (68, 69). Nevertheless, the mechanisms of ANS dysfunction and appearance of symptoms in CSVD patients require further study.

### Intervention Strategy on HRV After AIS

Many studies have reported elevated SNS activity and relatively suppressed PNS activity after AIS onset (39, 70). The stress response regulation of ANS and alterations in ANS activity play an essential role in the development of stroke. Thus, restoring the balance between SNS and PNS activity or readjusting HRV may be a novel strategy for management of stroke patients (Table 1).

**Table 1:** Intervention strategy on heart rate variability after acute ischemic stroke.

**Intervention Strategy**		**Studies**	**Research design**	**Participants**	**No. of patients**	**Main results**	**Conclusions**
Parasympathetic Activity Elevation	Transcutaneous electrical acupoint stimulation	Moreira et al. (71)	Observational study	Heart transplanted patients with age > 18.	22	The pNN50 and SDNN have been significantly improved on recovery (*p* < 0.05). The SDNN, LF, HF, LF/HF, and TP were significantly improved during TEAS (*p* < 0.05).	Transcutaneous electrical acupoint stimulation is an emerging strategy to enhance autonomic function of transplant patients.
	Intravenous electrical vagal nerve stimulation (VNS)	Khodaparast et al. (72)	Randomized controlled trial	Female Sprague-Dawley rats	17	Rats that received VNS during rehab significantly improved hit rate within the first week of therapy (+12.1 ± 2.2%, *p* < 0.001) and the second week compared with the first week (+5.2 ± 2.3%, *p* < 0.05).	VNS repeatedly paired with successful forelimb movements might be an efficient measure of motor rehabilitation in motor cortex ischemia.
		Kimberley et al. (73)	Randomized controlled trial	People implanted with the VNS device with a history of unilateral supratentorial ischemic stroke.	17	The clinically meaningful response rate of FMA-UE at day 90 was 88% with active VNS and 33% with control VNS (*p* < 0.05).	Patients with upper limb motor deficit after chronic stroke could benefit from VNS.
		Ay et al. (74)	Randomized controlled trial	A model of MCAO in rats.	32	The volume of ischemic damage was 41–45% smaller in animals receiving stimulation compared with the control group (*p* < 0.05).	VNS may reduce infarct volume and patients may have better neurological outcome after AIS.
		Garcia-Navarrete et al. (75)	Observational study	Patients with medication-resistant epilepsy.	43	Patients with generalized epilepsy had a reduction of 49.4 ± 34.8% in mean seizure frequency, those with focal epilepsy, 44.5 ± 35.6%, and those with temporal epilepsy, 63.0 ± 34.8%.	VNS could be an effective clinical treatment for medication-resistant epileptic patients.
SNS Suppression	Catestatin	Wang et al. (76)	Randomized controlled trial	Male Sprague-Dawley rats.	65	The chronic administration of Catestatin significantly increased SDNN, LF and HF and decreased LF/HF ratio (*p* < 0.01 for all) compared to the MI group.	Catestatin exerted cardio-protection by down-regulation of SNS.
	β-blockade, like metoprolol	Bieber et al. (77)	Randomized controlled trial	Male C57BL/6J mice.	177	Right-side tMCAO-treated mice showed a significant difference in LVEF and an increase in LV end-systolic and end-diastolic volumes (*p* < 0.001; ejection fraction: sham, *p* < 0.05; tMCAO [left], *p* < 0.01; ctrl, *p* < 0.001) from which protected by Metoprolol treatment (*p* < 0.05; end-systolic volume, *p* < 0.01; end-diastolic volume, *p* < 0.001).	Treatment of β-blockade may prevent the development of chronic cardiac dysfunction.
External Counter Pulsation (ECP)		Xiong et al. (78)	Randomized controlled trial	Patients with unilateral ischemic stroke within 14 days of stroke onset and healthy controls.	62	LF remained higher than baseline in the right-sided stroke patients after ECP (*p* = 0.050). TP also increased after ECP compared with baseline both in the left-sided and the right-sided stroke patients (*p* = 0.029; *P* = 0.017, respectively).	ECP may improve the sympathovagal balance in patients with subacute ischemic stroke.
Traditional Chinese Medicine (TCM)	Acupuncture	Yang et al. (79)	Randomized controlled trial	Male spontaneously hypertensive rats (SHRs) and WKY rats.	40	Compared with Non-Acupuncture group, the MBP was significantly decreased (*p* < 0.01) after 7 days of acupuncture. In the 14 days of acupuncture, SDNN and LF/HF significantly increased (*p* < 0.01).	Acupuncture decreased the increased blood pressure via the downregulation of renal sympathetic activity.
	Tele-acupuncture	Wang et al. (80)	Observational study	Chinese post-stroke patients (15 f, 14 m; mean age ± SD 64.7 ± 11.3 years; range 40–80 years).	29	HRV increased significantly (*p* < 0.05) during and 5–10 min after acupuncture. LF/HF changed markedly during treatment (*p* < 0.05).	Acupuncture could improve autonomic nervous function in the post-stroke patients.
	Moxibustion	Shin et al. (81)	Randomized controlled trial	Patients with prehypertension or stage I hypertension.	45	A significant decrease was found in SBP and DBP from baseline to 4 weeks of treatment (3 sessions/week) (MD −9.55; *p* = 0.0225, MD −7.55; *p* = 0.0098, respectively).	Moxibustion could lower blood pressure in patients at prehypertension stage.
Quitting Smoking		Murgia et al. (82)	Observational study: CHRIS	Participants from the CHRIS study.	4,751	Current smokers had higher HRV levels than never smokers: +0.091 (95%CI: 0.038, 0.144) log(SDNN) and +0.114 (95%CI: 0.043,0.183) log(RMSSD). Furthermore, each additional 10 g of tobacco daily smoked corresponded to −0.089 (95%CI: −0.124, −0.054) log(SDNN) (Figure 2) and −0.080 (95%CI: −0.126. −0.033) log(RMSSD).	Current progressively heavier smoking is suggested as an independent risk factor for a systemic dysautonomic effect. Smoking cessation could improve ANS function.
		Sumartiningsih et al. (83)	Randomized crossover study	Young adult male smokers (mean age 23 years) with a smoking habit of at least two years.	24	A significant difference of SDNN during exercise was found between groups C (control) and 3TC (3 mg nicotine of tobacco cigarettes) (*p* = 0.012), and between C and 3EC (3 mg nicotine of e-cigarettes) (*p* = 0.011).	Tobacco cigarettes smoking has a negative influence on heart rate response and exercise performance.
		Harte et al. (84)	Observational study	Healthy adult male (age 23–60 years) with a history of long-term smoking.	62	Successful quitters showed higher HRV compared to unsuccessful quitters at follow-up (SDNN, *p* = 0.05, d = 0.53; RMSSD, *p* = 0.01, d = 0.68; pNN50, *p* = 0.05, d = 0.50; LF power, *p* = 0.05, d = 0.51; HF power, *p* = 0.01, d = 0.73).	Smoking cessation significantly improved HRV in chronic male smokers.
		Bodin et al. (85)	Randomized controlled trial	Healthy people with high hostility levels (20–45 years, BMI ≤32 kg/m2).	149	lnHF was reduced by 0.31 ms^2^ (*p* = 0.04) when smokers reported having recently smoked cigarettes. The 24-h HF was significantly lower in smokers (mean = 5.24 ± SD = 0.14 ms^2^) than non-smokers (5.63 ± 0.07 ms2; *p* = 0.01).	Cigarette smoking could attenuate cardiac vagal regulation.
Abandoning Abuse of Alcohol		Wood et al. (86)	Meta-analysis of three trials: ERFC, EPIC-CVD, UK Biobank.	Current drinkers without previous cardiovascular disease.	599,912	Alcohol consumption was linearly associated with a higher risk of stroke (HR per 100 g per week higher consumption 1.14, 95% CI, 1.10–1.17), coronary disease excluding MI (1.06, 1.00–1.11), heart failure (1.09, 1.03–1.15), fatal hypertensive disease (1.24, 1.15–1.33); and fatal aortic aneurysm (1.15, 1.03–1.28).	The threshold of alcohol drinking for lowest risk of all-cause mortality was about 100 g/week.
Exercise	Exercise and fitness	Ricca-Mallada et al. (87)	Randomized controlled trial	Patients with CHF and LVEF ≤40% under complete evidence-based pharmacological treatment.	40	The training group showed an obvious increase in HF (*P* < 0.05) and RMSSD (*P* < 0.0288) after 24 weeks of training. 150 ms^2^/Hz for HF and 20 ms for RMSSD were the cut-off points predictive of an improvement in cardiac function and a reduction in adverse clinical events (AUC = 0.91, SE 0.09; CI 0.83–0.99) with high sensitivity of 0.85 and specificity of 0.80 (*p* = 0.01).	Exercise training effectively improves clinical outcomes in patients with low-risk chronic heart failure, and HRV is a valid tool to determine who will benefit most greatly.
		Rominger et al. (88)	Observational study	Healthy participants (mean age = 23.07 years; SD = 3.48 years).	97	Participants with more exercise performed better in the creative thinking task with greater relative HRV [β = 0.40, *t*(93) = −2.73, *p* = 0.008].	Regular exercising and fitness are correlated with better cognitive process and cardiac autonomic modulation.
	Yoga	Patil et al. (89)	Randomized passive-controlled trial	Non-diabetic offspring of type-2-diabetes parents (mean-age: 25.17 years).	64	Significant decrease in LF (*p* = 0.005), LF/HF ratio (*p* = 0.004), IR (*p* < 0.001), OGTT (*p* = 0.003) and increase in HF (*p* = 0.022) were found in yoga group participants.	Yoga can mitigate the risk of development of diabetes in offspring of diabetes parents.
		Christa et al. (90)	Randomized controlled trial	Patients post-MI	80	Higher HF power (*p* = 0.005) and TP (*p* = 0.01) were showed in the yoga group	Yoga could shift sympathovagal balance toward parasympathetic predominance.
	Chinese Tai Chi	Liu et al. (91)	Randomized controlled trial	Older individuals with depression score ≥10 (the Geriatric Depression Scale, GDS).	60	Depression measured by the GDS was significantly negatively associated with HF (*p* < 0.01), and positively associated with VLF (*p* < 0.05). Tai Chi group showed significant reductions in depression and LF (*p* < 0.05)	Chinese Tai Chi may alleviate depression in the elderly and present beneficial effect on regulation of ANS.
Psychological Adjustment		Hohl et al. (92)	Randomized controlled trial	Patients diagnosed with major depressive disorder (ICD-10)	11	It showed significant main effect of time with lower scores for anxiety [*F*_(110)_ = 37.57, *p* < 0.001, ω = 0.9], other feelings [*F*_(110)_ = 22.64, *p* = 0.001, ω = 0.8], physical sedation [*F*_(110)_ = 10.72, *p* = 0.008, ω = 0.7], significantly lower HR [*F*_(110)_ = 9.66, *p* = 0.01, ω = 0.7] and higher HF [*F*_(110)_ = 7.58, *p* = 0.02, ω = 0.6] after massage therapy sessions	Psychological consultation or intervention for depression or anxiety are useful strategies for both controlling of mental problem and positive regulation of HRV.

*VNS, intravenous electrical vagal nerve stimulation; FMA-UE, Fugl-Meyer assessment–upper extremity; Mean RR, mean of all normal RR intervals; pNN50, percentage of normal RR intervals that differed by more than 50 ms from the adjacent interval; SDNN, standard deviation of normal RR interval; RMSSD, root mean square of successive differences; LF, low-frequency; HF, high-frequency; LF/HF, the ratio of LF to HF power; VLF, very low-frequency; TP, total power; AIS, acute ischemic stroke; MI, myocardial infarction; LV, left ventricular; BNP, brain natriuretic peptide; tMCAO, transient middle cerebral artery occlusion; MBP, mean blood pressure; NE, norepinephrine; E, epinephrine; SD, standard deviation; HR, heart rate; HRV, heart rate variability; SBP, systolic blood pressure; DBP, diastolic blood pressure; MD, mean difference; CI, Confidence Interval; LVEF, left ventricular ejection fraction; CHF, chronic heart failure; NYHA, New York Heart Association Functional Class; 6mWT, 6-min walk test; AUC, the area under the curve; ln, natural logarithm; GDS, the geriatric depression scale*.

#### Elevated PNS Activity

The vagus nerve innervates the heart (93). Heart failure ultimately leads to a requirement for heart transplantation (94). Because of the parasympathetic denervation of implanted hearts, impaired HR control by the ANS increases metabolic demand, leading to a lower quality of life. A classic research showed no significant change in the resting coronary blood flow velocity in heart transplant patients after transcutaneous electrical nerve stimulation, indicating that the mechanism was at the microcirculatory level (95). Nonetheless, in a recent study, transcutaneous electrical acupoint stimulation was suggested as a potential strategy to enhance autonomic function of transplant patients (71). The effect of neurostimulation on heart transplant patients is uncertain and still need further explanations.

Inverse associations of PNS activity indexed by HRV and stroke, suggestive of altered vagal function, are important in the management of stroke. Oxidative stress, inflammation, and hypoxia (related to excessive SNS vasoconstriction) play pivotal roles in the occurrence and development of many chronic clinical conditions, including stroke (96). Biologically, the vagus nerve can inhibit SNS activity, oxidative stress, and inflammation (97), suggesting that stroke patients may benefit from vagal nerve activation. For example, electrical invasive vagal nerve stimulation (VNS) was reported to reduce food craving and body-weight in rats (98). Non-invasive devices for stimulation of the vagus nerve were also shown to decrease inflammation (99). Recently, VNS repeatedly paired with forelimb movements enhanced motor rehabilitation ability after motor cortex ischemia in rats, suggesting potential for stroke rehabilitation (72). Furthermore, a blinded randomized pilot study assessed the safety, feasibility, and potential effects of VNS paired with rehabilitation, and showed a benefit for patients with upper limb motor deficit after chronic stroke (73). VNS was also shown to reduce infarct volume and improve neurological outcome at 1 day after AIS in MCAO rats (74).

The mechanism of protection with VNS may involve a reduction in extracellular glutamate and reduced excitotoxicity during cerebral ischemia, and/or a reduction in inflammation and release of norepinephrine. PNS activation also increases cerebral blood flow and enhance neurogenesis (100). Interestingly, VNS was reported to be an effective clinical treatment for medication-resistant epileptic patients and depressed patients (75, 101). However, clinical testing is still required to determine the efficacy of VNS in patients with cerebrovascular disease, as serious adverse cardiovascular effects are possible with VNS (93). In summary, PNS activation can improve clinical outcomes, and may represent a new direction for prevention and therapy of stroke-related ANS dysfunction.

#### Suppression of SNS Activity

Over-activation of the SNS occurs in stroke patients. Elevated catecholamine levels increase the sympathoadrenal tone, resulting in hypertension and serious cardiovascular complications, while the peptide catestatin can protect the heart from excessive sympathetic drive (102). For example, chronic catestatin treatment reduced cardiac injury following myocardial infarction in rats, which was related to suppression of cardiac sympathetic activity and abnormal ANS function (76). Thus, application of sympatholytic agents may be useful for preventing cardiac complications after stroke by modulating the sympathetic-vagal balance.

β-blockade can also suppress SNS overactivation (77). For example, at 8 weeks after transient MCAO in mice, echocardiography and hemodynamic measurements showed that increased sympathetic activity, manifesting as decreased left ventricular ejection fraction and increased left ventricular volume, was a causative factor for development of chronic cardiac dysfunction (77). Furthermore, β-blockade with metoprolol prevented the development of chronic cardiac dysfunction related to chronic autonomic dysfunction, via deceleration of cardiac remodeling and inhibition of sympathetic signaling. If these findings can be confirmed clinically, this may provide a new therapeutic strategy to prevent cardiac dysfunction with early anti-sympathetic treatment.

#### External Counter Pulsation

External counter pulsation (ECP) is a non-invasive method used to enhance cerebral perfusion in patients with ischemic stroke, involving applied electrocardiography-triggered diastolic pressure created by air-filled cuffs on the lower extremities (103). The clinical efficacy of ECP was assessed by comparing beat-to-beat HRV in stroke patients with healthy controls (78). Results showed an increased beat-to-beat HRV after ECP in subacute ischemic stroke patients, whereas the LF R-R interval increased in patients with right-sided stroke, demonstrating improved sympathetic and parasympathetic tone after ECP, and suggesting that vagal activity influenced by arterial stiffness is a potential mechanism.

#### Traditional Chinese Medicine

Acupuncture or moxibustion is an important part of traditional Chinese medicine, and can be used as a complementary therapy for various diseases. For example, acupuncture was reported to decrease elevated blood pressure in animal models, via downregulation of renal sympathetic activity (79). Furthermore, tele-acupuncture was reported to significantly increase HRV during and for 5–10 min after acupuncture in post-stroke patients, and there was a marked improvement in the balance of SNS/PSN activity (80). Moxibustion may also lower blood pressure in patients with prehypertension (81), although the mechanisms involving regulation of SNS/PNS balance require further study.

#### Quitting Smoking

Smoking plays an important role in arteriosclerosis, and even second-hand smoke exposure can decrease HRV and increase the risk of cerebrovascular disease (104, 105). However, despite experimental studies showing negative effects of smoking on changes in HRV, population-based studies show conflicting results. Recently, a population-based epidemiological study reported that heavier smoking intensity was gradually detrimental to HRV, while smoking cessation increased HRV levels, suggesting that heavy smoking is an independent risk factor for a systemic dysautonomic effect (82). In addition, tobacco cigarette smoking has a negative influence on physiological responses and exercise performance, characterized by a significantly attenuated exercise-induced HR response and altered HRV (83). A distinct increase in BP and impaired cardiac function, with concomitant abnormal inflammation and endothelial dysfunction, which were regulated by the ANS, was observed with chronic smoking (106). Furthermore, smoking cessation significantly improved HRV in chronic male smokers (84). Finally, cigarette smoking attenuated cardiac vagal regulation, characterized by a lower high-frequency HRV, which may a pathophysiological mechanism of smoking (85). Thus, quitting smoking may be helpful for improving ANS function in smokers.

#### Stopping Alcohol Abuse

A number of prospective studies have shown that moderate intake of alcohol (~1–2 drinks per day) is associated with a lower incidence of stroke and myocardial infarction compared with no alcohol intake. Nevertheless, these data do not necessarily suggest that moderate alcohol intake is protective against either condition. High alcohol intake is undoubtable dangerous (86). For example, patients with alcohol use disorders show lower HRV compared with non-drinkers, while chronic, heavy alcohol use damaged the regulative capability of cardiac ANS function, and resting HRV was recovered or improved following at least 4 months of alcohol abstinence (107). Thus, stopping alcohol abuse may improve HRV abnormalities.

#### Exercise

Exercise can improve the ANS balance. In patients with chronic heart failure, exercise training significantly elevated HRV parasympathetic indices (HF and RMSSD) compared with non-exercise training (87). ANS dysfunction in parallel with cognitive impairment is common in patients post stroke. Indeed, post stroke patients had worse performance in the serial-3 subtraction task, and lower HRV, compared with healthy controls (108). Regular exercising and fitness are also correlated with cognitive process and cardiac autonomic modulation, in accordance with the theory that changes in cardiac responses to physical challenges may generalize to mental challenges. More regular exercise is associated with generation of more original ideas and larger increases in HRV (88). Various kinds of meditation and yoga can also increase HRV (109). For example, a marked increase in cardiac autonomic function and insulin resistance was found after 8 weeks of yoga training, suggesting that yoga can mitigate the risk of developing diabetes in the offspring of diabetic parents (89). For myocardial infarction patients, a 12-week yoga-based rehabilitation program increased overall HRV by shifting the sympathovagal balance toward parasympathetic predominance (90). Furthermore, Chinese Tai Chi has beneficial effects on regulation of the ANS (91). Thus, physical exercise is useful for improving HRV.

#### Psychological Adjustment

Stroke patients can exhibit a range of psychological problems including depression or anxiety, which have negative effects on HRV. Therapeutic communication, attentive care, and hand pressure during massage can improve HRV in major depressive disorders (92). Psychological consultation or intervention for depression or anxiety are useful strategies for controlling mental problems and positive regulation of HRV.

## Conclusions

Modulated by the ANS, various stressors incorporating acute and chronic risk factors for ischemic stroke can continuously and cumulatively affect the body, and are reflected by the level of sympathetic-vagal function activity during the stress response process. Decreased HRV is a precise and valid measurement of ANS, and has been confirmed to be correlated with different risk factors, characteristics of infarction, and poor clinical outcome in AIS patients. HRV may also be a predictor for development of CSVD, and over-activation of sympathetic tone may be a pathophysiologic mechanism. Although further studies are required, it is clear that ANS function plays an important role. Thus, HRV may be a more precise and efficient index for risk stratification and prognosis evaluation in stroke patients.

Based on several animal models of acute cerebral ischemia, therapeutic adjustment of ANS following HRV abnormalities can reduce cerebral and cardiac dysfunction, suggesting that sympathetic antagonism or parasympathetic activation at an early stage of injury may be a novel preventive and therapeutic strategy for patients with stroke. As early prevention and outcome improvement is important in stroke patients, further studies on the clinical implications of HRV in stroke are essential.

## Author Contributions

MZ was responsible for writing of the manuscript. LG conceived the study design and provided critical review of the manuscript. YW conceived the study design and provided critical review of the manuscript. All authors approved the final version of the manuscript.

### Conflict of Interest

The authors declare that the research was conducted in the absence of any commercial or financial relationships that could be construed as a potential conflict of interest.
